# High-Efficiency Polariton Organic Photodetectors via Trap-Assisted Photomultiplication

**DOI:** 10.3390/mi16121372

**Published:** 2025-12-01

**Authors:** Jui-Fen Chang, Sung-Jung Lin, Yang-Ching Huang, Sheng-Ping Lin

**Affiliations:** Department of Optics and Photonics, National Central University, Taoyuan 320317, Taiwan; curryjames0713@gmail.com (S.-J.L.); yangjing001102@gmail.com (Y.-C.H.); p0970970225@gmail.com (S.-P.L.)

**Keywords:** organic photodiodes, ultrastrong light–matter coupling, photomultiplication

## Abstract

We report a high-performance photomultiplication-type organic photodetector (OPD) based on a poly[2-methoxy-5-(3,7-dimethyloctyloxy)-1,4-phenylenevinylene]:[6,6]-phenyl-C_61_-butyric acid methyl ester (MDMO-PPV:PC_61_BM) active layer operating in the ultrastrong coupling regime. Systematic optimization of the PC_61_BM ratio in reference non-cavity devices confirms that trap-assisted hole injection from the Ag contact enables external quantum efficiencies (*EQEs*) exceeding 2000% and fast transient responses under 521 nm illumination, close to the absorption peak of MDMO-PPV. Incorporation of the optimized PC_61_BM ratio into a λ/2 microcavity produces well-resolved lower (LP) and upper (UP) polariton branches with a pronounced Rabi splitting of approximately 0.9 eV, confirming the establishment of ultrastrong light–matter coupling. The resulting cavity OPD exhibits a distinct wavelength-dependent response compared with its non-cavity counterpart, achieving maximum *EQEs* of 838% at 450 nm (near the UP mode) and 445% at 628 nm (corresponding to the LP mode). These spectral responses are attributed to cavity-induced field modulation, which enhances exciton generation beyond the primary absorption band of MDMO-PPV. Overall, this work demonstrates that combining photomultiplication mechanisms with cavity-field engineering provides an effective strategy for realizing narrowband, high-gain polaritonic photodetectors that surpass the spectral response limitations of conventional organic semiconductors.

## 1. Introduction

Extensive studies on organic polaritonic systems have established that strong and ultrastrong light–matter coupling can profoundly modify the electronic structure and wavefunction distribution of molecular materials through the formation of hybrid exciton–photon quasiparticles known as polaritons [1,2,3]. This hybridization fundamentally alters optical spectra, energy dispersion, carrier transport, and excited-state dynamics, thereby enabling unconventional functionalities in optoelectronic devices such as light-emitting diodes [4,5,6,7,8], lasers [9,10,11], transistors [12], and photodetectors [13,14]. In contrast to inorganic semiconductors, which host weakly bound Wannier–Mott excitons, organic semiconductors exhibit tightly bound Frenkel excitons with large oscillator strengths—characteristics that facilitate strong and even ultrastrong coupling at room temperature.

In 2018, pioneering research by Kéna-Cohen et al. [13] reported the first ultrastrongly coupled organic photodiode (OPD), where a SuPc/C_60_ blend embedded within a metallic microcavity exhibited a Rabi splitting exceeding 40% of the exciton energy. The resulting lower polariton (LP) branch extended the optical absorption beyond 1000 nm, leading to an external quantum efficiency (*EQE*) of 1.5% near the material’s intrinsic absorption edge. Importantly, the operation of such polaritonic OPDs differs fundamentally from conventional non-cavity counterparts. In non-cavity OPDs, excitons generated in the p-type absorber must diffuse to the donor-acceptor heterojunction within their limited diffusion length (~10 nm) to undergo charge separation, rendering nanoscale morphology a critical performance determinant [15]. In contrast, in polaritonic OPDs, the delocalized polariton wavefunction effectively extends the exciton diffusion length [16], facilitating transport toward the heterojunction. However, this delocalization also introduces competing effects: electron transfer kinetics are slowed, and the inherently short polariton lifetime (~1 ps) imposes an additional constraint. Ultrafast spectroscopy by Scholes and co-workers further revealed that electron transfer from polaritons in prototype poly(3-hexylthiophene):[6,6]-phenyl-C_61_-butyric acid methyl ester (P3HT:PC_61_BM) heterojunctions can occur within ~1 ps, driven by the high electron affinity of fullerenes—comparable to the polariton lifetime itself [17]. These results emphasize the importance of achieving an optimal balance between polariton transport and charge-transfer dynamics in polaritonic OPDs, which requires the rational design of material systems and device architectures that simultaneously enhance conductivity and optimize heterojunction interfaces.

Despite these advances, most polaritonic OPDs still exhibit relatively low optoelectronic conversion efficiencies. Although cavity-induced mode splitting can extend spectral response beyond the intrinsic absorption band edge, overall *EQEs* remain significantly below those of non-cavity devices, largely due to mode-selective absorption and optical losses associated with the cavity mirrors. Moreover, nearly all reported polaritonic OPDs employ bilayer or bulk-heterojunction geometries analogous to those in organic photovoltaics, where photocurrent generation arises from polariton dissociation into oppositely directed electron and hole currents—a process inherently constrained to *EQEs* below unity.

To address these limitations, we introduce a trap-assisted photomultiplication mechanism into strongly coupled OPDs, functioning analogously to Schottky photodiodes. In this architecture, hole injection mediated by trapped electrons induces photocurrent gain for each absorbed photon, effectively surpassing the unity-efficiency limit associated with conventional polariton dissociation processes [18,19,20]. The active layer comprises a strongly coupled p-type conjugated polymer lightly doped with fullerene derivatives, which provides high hole conductivity and robust light–matter coupling while minimizing unwanted electronic perturbations. In addition, the delocalized nature of polaritons enables long-range transport toward the electrodes, facilitating exciton dissociation and the formation of interfacial electron traps that promote efficient hole tunneling and current amplification. Collectively, this strategy establishes a new design paradigm for high-gain polaritonic OPDs that merge strong coupling physics with photoconductive amplification.

## 2. Device Fabrication and Characterization Method

**Figure 1:**
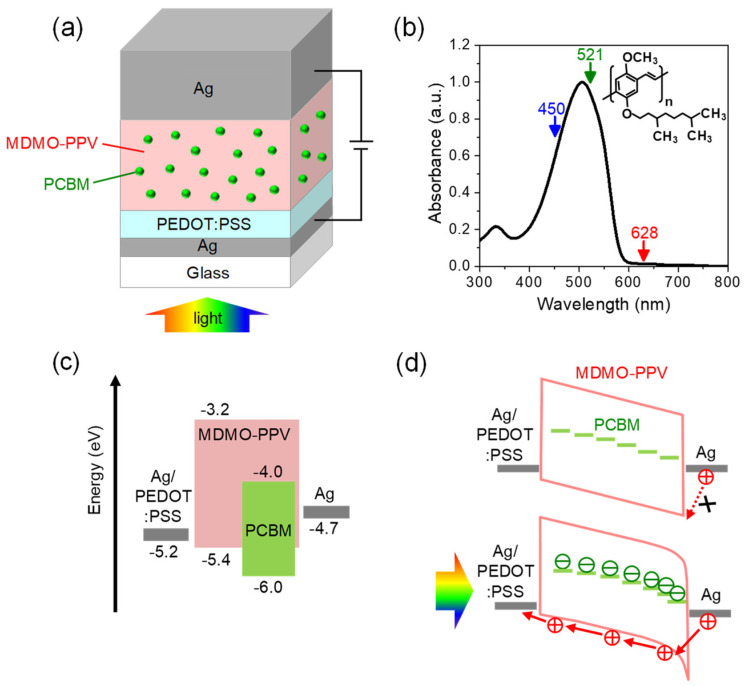
(**a**) Schematic illustration of the cavity OPD based on an MDMO-PPV:PC_61_BM active layer. (**b**) Absorption spectrum of the MDMO-PPV film, with arrows marking the specific wavelengths of the LEDs employed for photocurrent measurements. (**c**) Energy level diagram of the materials used. (**d**) Operation mechanism of the OPD under dark and illuminated conditions.

The device architecture of the photomultiplication-type OPD is schematically illustrated in Figure 1a. It features a *λ*/2 optical microcavity sandwiched between two reflective silver (Ag) electrodes. The bottom Ag electrode (20–30 nm) serves as a semitransparent illumination window, whereas the top Ag electrode (~150 nm) acts as the hole-injecting contact under positive bias. A thin PEDOT:PSS interlayer was spin-coated onto the bottom Ag surface to reduce surface roughness and to fine-tune the optical thickness for cavity resonance. The active layer consists of the conjugated polymer poly[2-methoxy-5-(3,7-dimethyloctyloxy)-1,4-phenylenevinylene] (MDMO-PPV, M_w_~100 kDa; purchased from 1-Material Inc., Dorval, QC, Canada), which serves as both the strongly coupled absorber and the hole-transporting medium. MDMO-PPV was selected for its strong optical absorption, enabling ultrastrong light–matter coupling [6]. The absorption spectrum and optical constants (*n*, *k*) of the MDMO-PPV film are shown in Figure 1b and Supplementary Materials, Figure S1, respectively. The film exhibits a pronounced absorption band centered at 504 nm, corresponding to a peak absorption coefficient of 4π*k*/*λ* = 2.1 × 10^5^ cm^−1^. Furthermore, as shown in the energy-level diagram in Figure 1c, the relatively deep highest occupied molecular orbital level of MDMO-PPV (approximately −5.4 eV) facilitates the formation of a suitable Schottky barrier with the top Ag electrode. To induce photomultiplication, a small fraction of the fullerene derivative [6,6]-phenyl-C_61_-butyric acid methyl ester (PC_61_BM; purchased from Lumtec, New Taipei City, Taiwan) was incorporated into the polymer matrix as electron-trapping centers. The MDMO-PPV:PC_61_BM blend film was deposited by spin-coating from a chlorobenzene solution, producing a uniform active layer approximately 70 nm thick. Reference non-cavity OPDs were also fabricated on ITO substrates to investigate the photomultiplication mechanism. The active area of all OPDs was 2 × 2 mm^2^.

The operating principle of the photomultiplication processes is depicted in Figure 1d. In the dark state, the Ag/MDMO-PPV:PC_61_BM interface imposes a hole-injection barrier, thereby suppressing the dark current. Upon photoexcitation, excitons dissociate at dispersed PC_61_BM trapping sites near the electrode. The resulting trapped electrons locally modulate the electric field, effectively lowering the hole-injection barrier and enabling field-assisted tunneling. This process produces a photocurrent gain that exceeds the unity quantum efficiency limit.

In this study, all optoelectronic characterizations of the OPDs were carried out using an Agilent B1500A semiconductor parameter analyzer (Santa Rosa, CA, USA). Photocurrent responses were measured under illumination from the bottom Ag or ITO electrode side using monochromatic LEDs at 450 nm, 521 nm, and 628 nm (incident power *P_in_* = 1 mW/cm^2^), corresponding to different regions within the MDMO-PPV absorption spectrum. The *EQE*, responsivity (*R*), and specific detectivity (D*) were calculated according to the following equations [21]:

$$EQE=\frac{J_{ph}h\nu }{P_{in}e}=\frac{\left(J_{light}-J_{dark}\right)h\nu }{P_{in}e}$$

$$R=\frac{J_{ph}}{P_{in}}$$

$$D^{*}=\frac{R}{\sqrt{2eJ_{dark}}}$$

where *J_ph_* represents the photocurrent density, *J_light_* is the current density under illumination, *J_dark_* is the dark current density, *h* denotes the Planck constant, *ν* is the frequency of the incident light, and *e* is the elementary charge. The transient response was measured using an oscilloscope connected in series with a 50 Ohms load resistor. All electrical measurements were conducted in a nitrogen glove box.

Additionally, the exciton generation rate *G*(z,λ) at depth z within the device and illumination wavelength λ was calculated as

$$G(z,\lambda )=\frac{\lambda }{hc}Q(z,\lambda )$$

$$Q(z,\lambda )=\frac{1}{2}c\varepsilon_{0}\alpha_{\lambda }n_{\lambda }{\left|E\left(z\right)\right|}^{2}$$

where Q(z,λ) denotes the average energy dissipated per second at position z. Here, *c* is the vacuum speed of light, $\varepsilon_{0}$ is the vacuum permittivity, $\alpha_{\lambda }$ and $n_{\lambda }$ are the absorption coefficient and real refractive index of the active layer at wavelength λ, respectively, and *E*(z) represents the spatial distribution of the electric field within the device. The field distribution *E*(z) was obtained through optical simulations performed using Essential Macleod (version V12.2).

## 3. Results and Discussion

**Figure 2:**
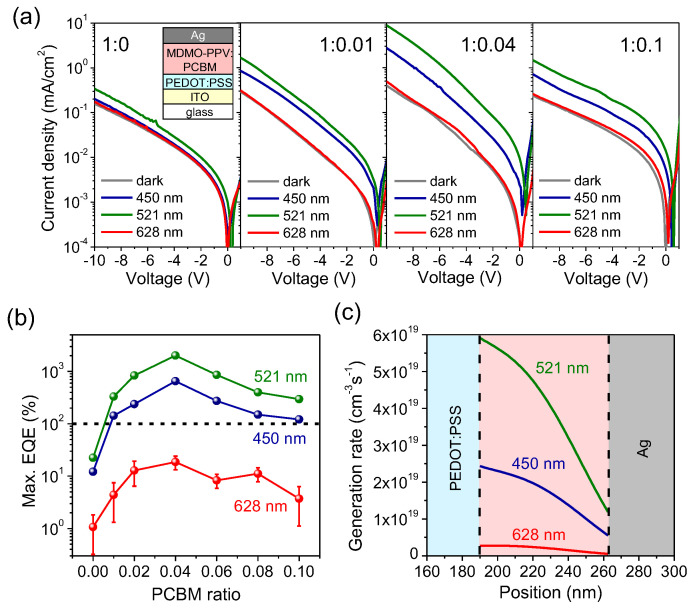
(**a**) *J*-*V* characteristics of reference non-cavity OPDs with varying PC_61_BM doping ratios in MDMO-PPV, measured in the dark and under illumination at selected wavelengths. (**b**) Maximum *EQE* at −10 V as a function of PC_61_BM ratio. (**c**) Calculated exciton generation rate profiles in the MDMO-PPV:PC_61_BM active layer under different excitation wavelengths.

Because trap-assisted photomultiplication depends critically on the concentration of the n-type dopant, a series of reference non-cavity OPDs was studied to decouple the effects of material composition and interfacial charge dynamics from those induced by the optical cavity. Figure 2a shows the *J*-*V* characteristics of representative non-cavity OPDs with PC_61_BM ratios of 0, 0.01, 0.04, and 0.1. The pristine MDMO-PPV device (without PC_61_BM) exhibits only weak photocurrent under 521 nm illumination and negligible response at 450 and 628 nm, with all maximum *EQEs* below 100% at −10 V (Figure 2b), indicating the absence of photomultiplication. Introducing a small PC_61_BM ratio of 0.01 markedly enhances the photocurrent at 450 and 521 nm and results in *EQEs* exceeding 100%, a clear indication of the onset of trap-assisted gain. Increasing the PC_61_BM ratio to 0.04 yields the optimal performance, whereas further doping leads to diminished photocurrent and *EQE*. This degradation arises from the formation of conductive electron pathways, which reduce effective electron trapping and thereby suppress trap-assisted hole injection in heavily doped devices [22]. Across all compositions, the strongest photoresponse consistently occurs under 521 nm illumination, close to the absorption maximum of MDMO-PPV. In contrast, excitation at 628 nm—within the absorption tail—produces minimal response in the absence of photomultiplication. This wavelength dependence can be quantitatively explained by the local exciton generation rate near the Ag/MDMO-PPV:PC_61_BM interface. As shown in Figure 2c, the calculated generation profiles confirm that in the relatively thin (~70 nm) MDMO-PPV:PC_61_BM layer, exciton generation is primarily dictated by the material’s intrinsic absorption spectrum, rather than optical field penetration effects that dominate in thicker devices [23]. Consequently, excitation at 521 nm, near the absorption peak, yields the highest exciton generation rate and *EQE*, whereas excitation at 628 nm within the absorption tail results in a substantially weaker response. Supplementary Materials, Figure S2a–c and Table 1 summarize the performance of the optimized non-cavity OPD with a PC_61_BM ratio of 0.04, demonstrating excellent photomultiplication characteristics. Under 521 nm illumination at −10 V, the device achieves *EQE* = 2019%, *R* = 8.47 A/W, and D* = 7.49 × 10^11^ Jones. It also maintains stable performance under repeated pulsed excitation (Supplementary Materials, Figure S2d), with rise and fall times of 18 ms and 26 ms, respectively. These rapid temporal responses indicate efficient electron trapping/release dynamics and fast hole transport through the thin active layer.

**Figure 3:**
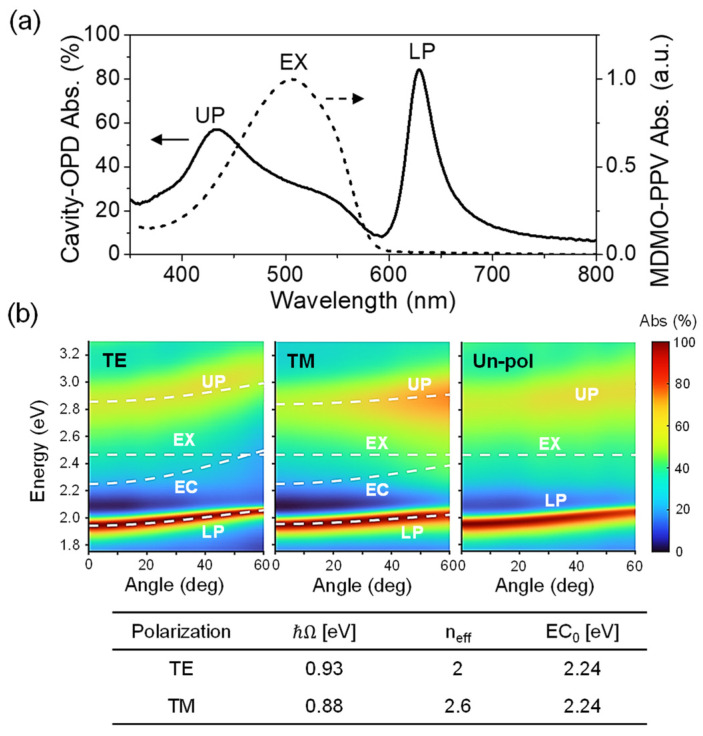
(**a**) Absorption spectrum of the cavity OPD incorporating an MDMO-PPV: PC_61_BM (1:0.04) active layer, compared with that of the pristine MDMO-PPV film. (**b**) Angle-resolved absorption spectra of the cavity OPD under TE, TM, and unpolarized illumination. The inset table lists the fitted parameters for the extraction of LP, UP, and EC mode dispersions.

Based on the optimized active-layer composition, a cavity OPD was fabricated incorporating an MDMO-PPV:PC_61_BM (1:0.04) blend film with a thickness of approximately 70 nm. The absorption spectrum (A), obtained from reflectance measurements at a 5° incidence angle (Supplementary Materials, Figure S3a), is shown in Figure 3a. Owing to the negligible transmittance (T ≈ 0) of the cavity structure, the absorption was approximated as A ≈ 1—Reflectance. The spectrum clearly reveals the LP and upper polariton (UP) modes flanking the exciton resonance (EX) of MDMO-PPV at 504 nm (2.46 eV), with the LP mode tuned to 628 nm within the polymer’s absorption tail. Angle-resolved absorption spectra under TE, TM, and unpolarized illumination are shown in Figure 3b. The measured absorption peaks were analyzed using the Hopfield Hamiltonian with a dispersionless EX mode to extract the dispersions of the LP, UP, and uncoupled cavity (EC) modes. The extracted parameters, including the Rabi splitting ℏΩ, effective refractive index n_eff_, and cavity mode energy at normal incidence EC_0_, are summarized in the inset of Figure 3b. The fitted Rabi splittings are 0.93 eV for TE and 0.88 eV for TM polarization, corresponding to approximately 38% and 36% of the bare exciton energy, respectively. These large coupling ratios (ℏΩ/EX > 20%) unambiguously confirm that the device operates in the ultrastrong coupling regime [24]. Furthermore, the Rabi splitting values closely match those observed in the pristine MDMO-PPV cavity device (Supplementary Materials, Figure S4), indicating that the incorporation of a small amount of PC_61_BM has a negligible impact on the coupling strength. Under unpolarized illumination, the spectra exhibit an averaged response of the TE and TM polarizations, consistent with realistic LED excitation conditions.

Overall, the ultrastrong coupling gives rise to a spectrally narrow and nearly dispersionless LP branch, exhibiting a maximum absorption exceeding 80% at normal incidence. This high absorption persists up to an incidence angle of 60°, with only a minor reduction at larger angles. In contrast, the UP absorption increases slightly with angle, reaching ~60% at 60°, yet remains consistently weaker than the LP mode. These angular trends reflect the evolving photonic fractions of the hybrid polariton states. As illustrated for TE polarization in Supplementary Materials, Figure S5, the LP mode is predominantly photon-like at small angles, whereas the UP mode becomes increasingly photonic at larger angles.

**Figure 4:**
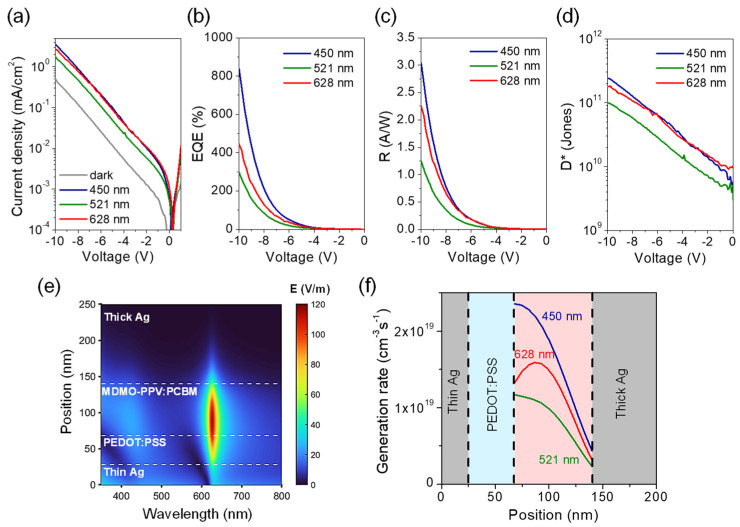
(**a**) *J*-*V* characteristics and corresponding (**b**–**d**) *EQE*, *R*, and D* of the cavity OPD incorporating an MDMO-PPV:PC_61_BM (1:0.04) active layer under illumination at selected wavelengths. (**e**) Simulated spatial distribution of the electric field intensity in the cavity OPD as a function of wavelength. (**f**) Calculated exciton generation rate within the MDMO-PPV:PC_61_BM active layer under different excitation wavelengths.

Figure 4a shows the *J*-*V* characteristics of the cavity OPD with a PC_61_BM ratio of 0.04, measured under the same illumination conditions as the non-cavity reference device. Unlike the reference, the cavity OPD exhibits a distinctly wavelength-dependent response, with the photocurrent maximized at 450 nm, minimized at 521 nm, and enhanced again at 628 nm. The best performance is obtained under 450 nm illumination, achieving *EQE* = 838%, *R* = 3.04 A/W, and D* = 2.39 × 10^11^ Jones at −10 V (Figure 4b–d). Notably, at 628 nm, corresponding to the LP mode, the EQE reaches 445% at −10 V, demonstrating efficient photomultiplication beyond the intrinsic absorption band of MDMO-PPV. A summary of the cavity OPD characteristics at selected illumination wavelengths is provided in Table 1. By comparison, the cavity OPD fabricated using only MDMO-PPV (without PC_61_BM) produces negligible photocurrent across all wavelengths due to the absence of photomultiplication (data not shown).

This unique spectral response, absent in the non-cavity device, arises from microcavity-induced modulation of the optical field. To verify this mechanism, the electric field distribution at normal incidence was simulated using the transfer-matrix method, fitted to the measured 5° reflectance spectrum (Supplementary Materials, Figure S3a). The simulations show pronounced field enhancement at both the LP and UP resonances, whereas the exciton mode exhibits much weaker field intensity (Figure 4e and Figure S3b). In particular, the LP mode generates an intensely localized field at 628 nm, effectively compensating for the weak intrinsic absorption of MDMO-PPV and thereby promoting exciton generation, leading to *EQEs* exceeding 100%. In contrast, excitation at 450 nm (near the UP mode) results in a somewhat weaker cavity field but benefits from the intrinsically stronger absorption of MDMO-PPV, thus producing the highest overall exciton generation rate and *EQE*. At 521 nm, near the EX mode, the cavity field intensity is minimal; therefore, despite the strong material absorption, the device exhibits the lowest *EQE*. The simulated spatial exciton generation profiles (Figure 4f) are in excellent agreement with the measured spectral photoresponses, confirming a strong correlation between the cavity-field distribution and the resulting photocurrent behavior. Supplementary Materials, Figure S6 further shows the transient response of the cavity OPD under 450 nm illumination at −10 V, with rise and fall times of 18 ms and 25 ms, respectively, which are nearly identical to those of the non-cavity device (see Table 1). This similarity indicates that the cavity structure exerts minimal influence on the transient behavior, likely because the device dynamics in this photomultiplication-type architecture are predominantly governed by hole injection and transport.

Together, these results highlight the central role of cavity-field engineering in governing photomultiplication efficiency. Achieving high gain requires maximizing both the field intensity and the spectral overlap between the polariton modes and the absorber’s optical transitions—particularly crucial for the LP mode near the band edge. The field intensity at a target wavelength (such as the LP mode) can be further increased by adjusting the cavity thickness to realize an ideal antireflection condition. As supported by additional simulations (Supplementary Materials, Figure S3a,c), implementing an antireflection design at the LP wavelength (628 nm)—by tuning the thicknesses of the thin Ag and MDMO-PPV:PC_61_BM layers such that the complex admittance trajectory approaches that of air [25]—can enhance the internal field near the Ag/MDMO-PPV:PC_61_BM interface by ~10%. Such enhancement offers a promising pathway for further improving the *EQE*.

## 4. Conclusions

In summary, we have demonstrated a highly efficient photomultiplication-type OPD with an MDMO-PPV:PC_61_BM active layer operating in the ultrastrong coupling regime. Studies on reference non-cavity devices show that introducing a low PC_61_BM ratio (0.04) in MDMO-PPV optimizes electron trapping and hole injection from the Ag contact, enabling *EQEs* exceeding 2000% and rapid transient responses under 521 nm illumination near the MDMO-PPV absorption maximum. Incorporating this optimized blend into a *λ*/2 microcavity produces well-defined LP and UP branches with a large Rabi splitting of ~0.9 eV, confirming that ultrastrong coupling is largely preserved upon PC_61_BM doping. Compared with the non-cavity reference, the cavity OPD exhibits a distinct spectral photoresponse, achieving an *EQE* of 445% at 628 nm (LP mode) and 838% at 450 nm (near the UP mode). Optical simulations attribute this wavelength-dependent behavior to cavity-induced field enhancement at the polariton resonances: the LP mode generates a strongly confined field that compensates for weak intrinsic absorption in the red tail, while the UP mode benefits from both enhanced cavity field and higher intrinsic absorption. These results highlight the effectiveness of combining photomultiplication mechanisms with cavity-field engineering to boost photodetection efficiency and extend spectral sensitivity. They further suggest that integrating antireflection microcavity designs could enhance the internal field near polariton resonances, providing a promising strategy for developing narrowband, high-gain polaritonic photodetectors beyond the intrinsic absorption limits of conventional organic semiconductors.

## Figures and Tables

**Table 1:** Summary of the maximum *EQE*, *R*, D* values for the optimized non-cavity and cavity OPDs incorporating an MDMO-PPV:PC_61_BM (1:0.04) active layer under selected illumination wavelengths, all measured at −10 V. The rise and fall times correspond to the wavelength at which the maximum *EQE* is achieved.

	Illumination *λ* (nm)	*EQE* (%)	*R* (A/W)	D* (Jones)	Rise/Fall Time (ms)
	450	653	2.37	2.09 × 10^11^	-
non-cavity OPD	521	2019	8.47	7.49 × 10^11^	18/26
	628	19	0.056	5.93 × 10^9^	-
	450	838	3.04	2.39 × 10^11^	18/25
cavity OPD	521	297	1.25	9.81 × 10^10^	-
	628	445	2.26	1.77 × 10^11^	-

## Data Availability

The original contributions presented in this study are included in the article. Further inquiries can be directed to the corresponding author.

## Supplementary Materials

The following supporting information can be downloaded at https://www.mdpi.com/article/10.3390/mi16121372/s1, Figure S1: Optical constants (*n*, *k*) of the MDMO-PPV film; Figure S2: Bias-dependent *EQE*, *R*, *D**, and transient response of the non-cavity OPD with an MDMO-PPV:PC_61_BM (1:0.04) active layer; Figure S3: Experimental and simulated reflectance spectra, and simulated electric field distributions of the cavity OPD with an MDMO-PPV:PC_61_BM (1:0.04) active layer under different excitation wavelengths; Figure S4: Angle-resolved absorption spectra of the cavity OPD containing pristine MDMO-PPV (without PC_61_BM) under TE and TM illumination; Figure S5: Excitonic and photonic fractions of the LP and UP modes for the cavity OPD with an MDMO-PPV:PC_61_BM (1:0.04) active layer under TE-polarized illumination; Figure S6: Transient response of the cavity OPD with an MDMO-PPV:PC_61_BM (1:0.04) active layer.
