# Supplementary material for: High-Efficiency Polariton Organic Photodetectors via Trap-Assisted Photomultiplication
Source: Micromachines (Basel). 2025 Dec 1;16(12):1372. doi: 10.3390/mi16121372 (PMC12735027; doi:10.3390/mi16121372)
Supplement: Supplementary file 1 [file micromachines-16-01372-s001.zip › micromachines-3989671-supplementary.pdf]

Supplementary Materials

High-Efficiency Polariton Organic Photodetectors via Trap-Assisted Photomultiplication

Jui-Fen Chang *, Sung-Jung Lin, Yang-Ching Huang, and Sheng-Ping Lin

Department of Optics and Photonics, National Central University, Zhongli 320317, Taiwan;

Correspondence: jfchang@dop.ncu.edu.tw

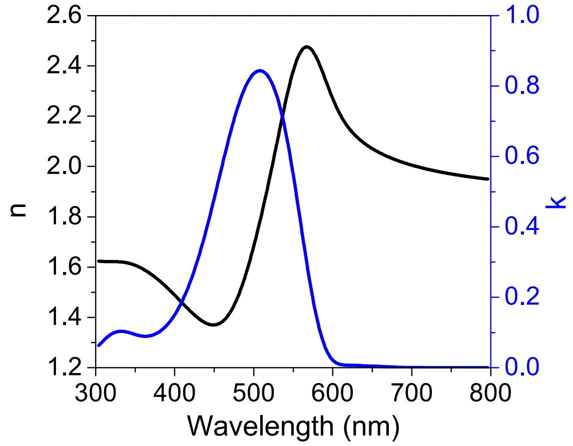

Figure S1. (*n*,*k*) spectra of MDMO-PPV film.

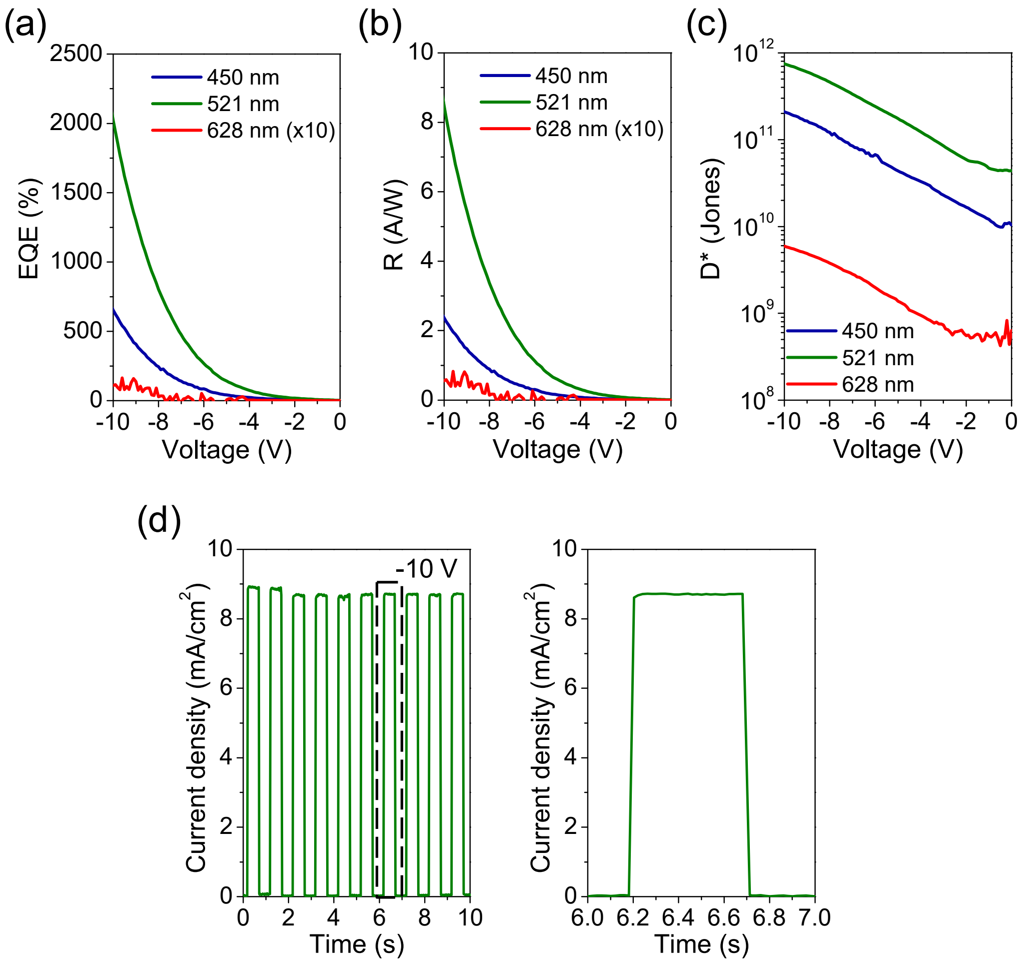

Figure S2. Bias-dependent (a) external quantum efficiency (*EQE*), (b) responsivity (*R*), (c) specific detectivity (*D**), and (d) transient response of the non-cavity OPD incorporating an MDMO-PPV:$PC_{61}BM$ (1:0.04) active layer. The transient response was measured at -10 V under 521 nm illumination.

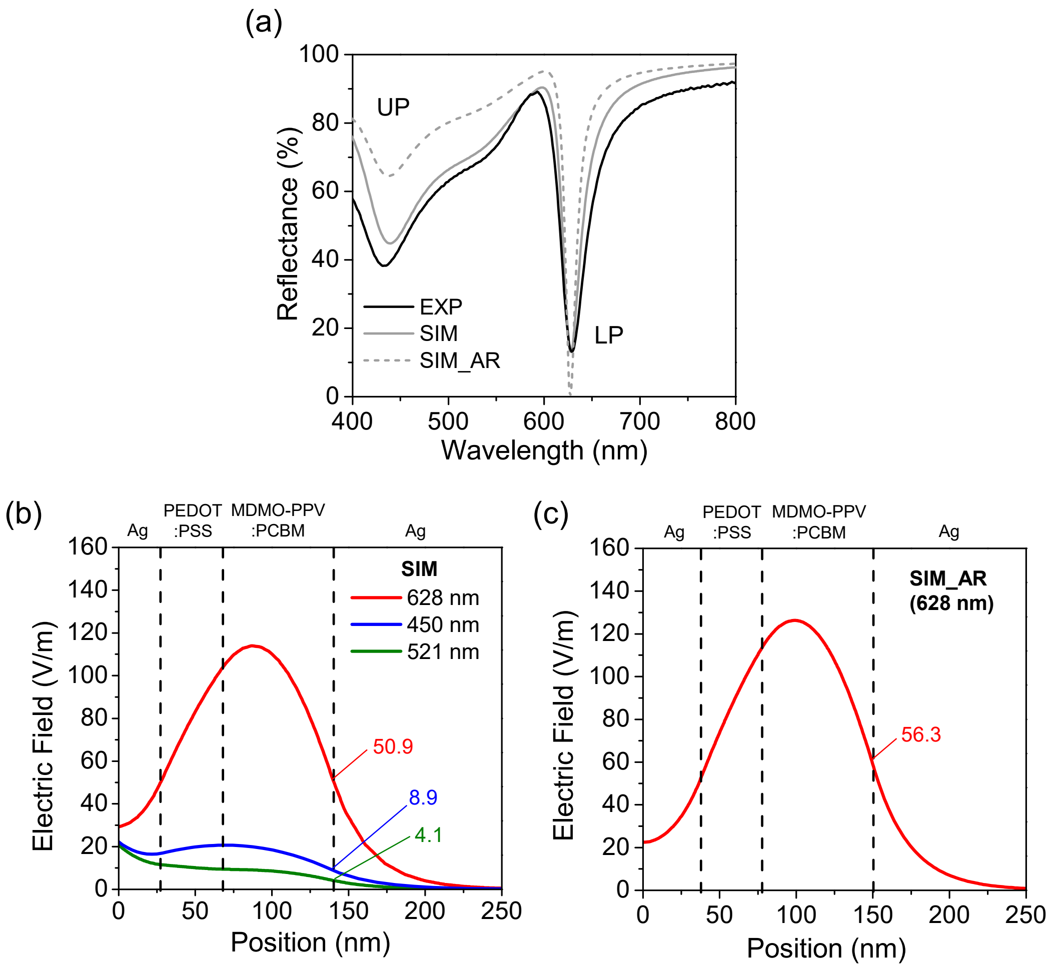

Figure S3. (a) Experimental reflectance spectrum of the cavity OPD incorporating an MDMO-PPV:$PC_{61}BM$ (1:0.04) active layer measured at a 5° incidence angle, compared with simulated spectra at normal incidence and for an optimized antireflection design at the LP mode (628 nm). (b) Simulated electric field distributions of the current device under various excitation wavelengths, with the field intensity at the Ag/MDMO-PPV:$PC_{61}BM$ interface highlighted. (c) Simulated electric field distribution of the optimized device with the antireflection design at 628 nm, showing enhanced field strength relative to the current device.

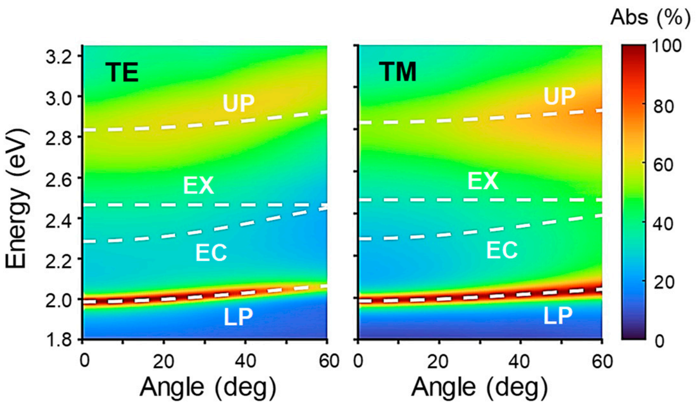

Polarization	$\hbar\Omega$ [eV]	n_{eff}	EC_0 [eV]
TE	0.88	2.4	2.285
TM	0.88	2.87	2.28

Figure S4. Angle-resolved absorption spectra of the cavity OPD containing pristine MDMO-PPV (without $PC_{61}BM$) under TE and TM illumination. The inset table lists the fitted parameters for the extraction of LP, UP, and EC mode dispersions.

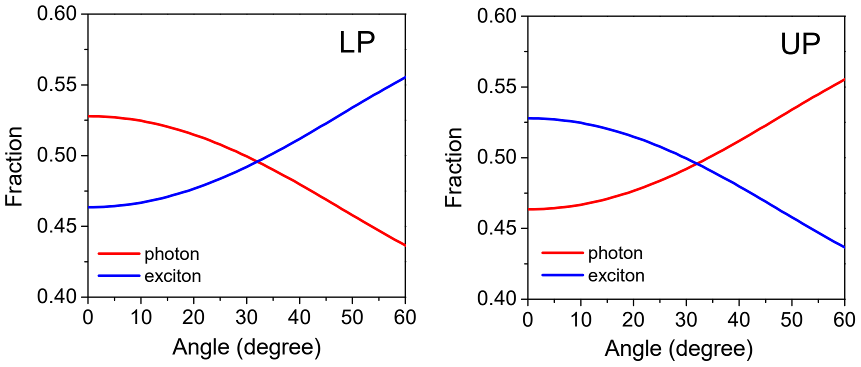

Figure S5. Excitonic and photonic fractions of the LP and UP modes, extracted from Hopfield Hamiltonian fitting of the cavity OPD with an MDMO-PPV:$PC_{61}BM$ (1:0.04) active layer under TE-polarized illumination. The results reveal pronounced light-matter hybridization across a broad angular range.

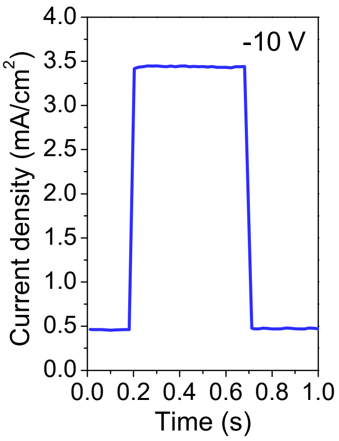

Figure S6. Transient response of the cavity OPD incorporating an MDMO-PPV:$PC_{61}BM$ (1:0.04) active layer, measured at -10 V under 450 nm illumination.
